# Ozonation of Human Blood Induces a Remarkable Upregulation of Heme Oxygenase-1 and Heat Stress Protein-70

**DOI:** 10.1155/2007/26785

**Published:** 2007-09-23

**Authors:** Velio Bocci, Carlo Aldinucci, Francesca Mosci, Fabio Carraro, Giuseppe Valacchi

**Affiliations:** Department of Physiology, University of Siena, 53100 Siena, Italy

## Abstract

Heme oxygenase-I (HO-1) has emerged as one of the most protective enzymes and its pleiotropic activities have been demonstrated in a variety of human pathologies. Unpublished observations have shown that HO-1 is induced after the infusion of ozonated blood into the respective donors, and many other experimental observations have demonstrated the efficacy of oxidizing agents. It appeared worthwhile to evaluate whether we could better define the activity of potential inducers such as hydrogen peroxide and ozonated human plasma. Human vascular endothelial cells at confluence were challenged with different concentrations of these inducers and the simultaneous production of nitric oxide (NO); and HO-1 was measured by either measuring nitrite, or bilirubin formation, or/and the immune reactivity of the protein by Western blot using a rabbit antihuman HO-1 and Hsp-70. The results show that production of both NO and HO-1 is fairly dose dependent but is particularly elevated using human plasma after transient exposure to a medium ozone concentration. At this concentration, there is also induction of Hsp-70. The results clarify another positive effect achievable by the use of ozone therapy.

## 1. INTRODUCTION

Heme oxygenase-1 (HO-1) has been defined as a cellular Hercules [1] because of its potent and pleiotropic biological activities. After Tenhunen et al., in 1972, published the first report describing the enzymatic degradation of heme [2], the interest in this enzyme has grown exponentially and to our knowledge, today there are almost 4000 publications on this topic. The induction of HO-1, mainly considered a generalized response to oxidative stress, results in an increased formation of carbon monoxide (CO) and bilirubin while redox-active iron is rapidly and safely sequestered by the simultaneous coinduction of ferritin [3]. Amazingly, bilirubin, thought to be always a useless and possibly a toxic molecule, has revealed to be an excellent lipophilic antioxidant, far more effective than reduced glutathione [4]. CO, a well-known deadly gas molecule, when released in trace amounts, appears to be cytoprotective because of its cyclic-GMP-mediated modulation of vascular tone and neurotransmission [5]. Thus, it is not surprising that HO-1 has been shown to prevent or improve pathological states associated with cardiomyopathy, chronic limb ischemia, hypertension, endotoxemia, organ transplantation, chronic infections, pulmonary diseases, diabetes, and autoimmune diseases [6–8]. In all of these cases, the initial etiological cause is perpetuated by a chronic oxidative stress that tends to accelerate the progression of the disease. Orthodox medicine has several valid drugs that, rather than “curing” the disease, aim to stabilize it. A supplemental administration of antioxidants is harmless, but it is of little help [9] because the main problem is the intracellular unbalance between an excessive production of reactive oxygen species (ROS), peroxidation products, and proinflammatory cytokines in front of the reduced efficiency of the antioxidant system.

During the last decade, our biological and clinical work [10–14] has shown that a judiciously performed ozone therapy in still responsive patients is able to correct this abnormal situation by upregulating antioxidant enzymes such as SOD, GSH-peroxidases, reductases and transferases, and glucose-6-phosphate dehydrogenase. This result, firstly demonstrated for SOD in 1996 [15], is due to the repetition of small and acute oxidative stresses induced by precise doses of well-calibrated ozone against the potent antioxidant capacity of human blood. This process, now universally present from bacteria to fungi, plants, and mammals, has been defined as an adaptation to acute oxidative stresses or oxidative preconditioning [10–12, 15, 16]. Moreover, this apparently paradoxical biological effect has been postulated [10–12] to be supported by the simultaneous induction of HO-1 because this enzyme is induced by hydrogen peroxide (H_2_O_2_), ultraviolet radiation, ROS [17], and by heme [6]. During the few minutes of ozonation of human blood ex vivo; ozone dissolves in the plasmatic water and generates messengers such as H_2_O_2_ and lipid oxidation products (LOPs) which, after the prompt reinfusion of the ozonated blood in the donor, are responsible of the several biological effects, of which an important one is the upregulation of the antioxidant system intuitively explained as a defensive reaction.

Owing to the fact that ozone therapy, besides behaving as a calculated acute oxidative stress, favors the release of a small amount of hemoglobin, it appears reasonable to envisage the subsequent induction of HO-1 in the donor patient. On this basis, we have performed the following preliminary study to ascertain whether potential HO-1 inducers can stimulate the synthesis of this enzyme in human endothelial cells.

## 2. MATERIALS AND METHODS

### 2.1. Chemicals

Sodium nitrite (NaNO_2_), H_2_O_2_ (30% solution), L-arginine, and the NO synthase inhibitor NG-nitro-L-arginine methyl ester (L-NAME) were purchased from Sigma Chemical and Aldrich Chemical (MO, USA). Hemin was dissolved in 10% ammonium hydroxide in 0.15 M NaCl to prepare a stock solution of 100 mg/mL and then further diluted 1 : 40 with sterile 0.15 M NaCl (2.5 *μ*g/1 *μ*L).

### 2.2. Ozone generation and measurement

Ozone was generated from medical-grade oxygen (O_2_) using electrical corona arc discharge, by the O_3_ generator (Model Ozonosan PM100K, Hansler. GmbH, Iffezheim, Germany), which allows the gas flow rate and O_3_ concentration to be controlled in real time by photometric determination, as recommended by the Standardisation Committee of the International O_3_ Association. The ozone flow rate was kept constant at 3 L/min in all experiments. Polypropylene syringes (ozone-resistant) were used throughout the reaction procedure to ensure containment of O_3_ and consistency in concentrations.

### 2.3. Collection of human blood and plasma samples

Blood samples were taken from one of us in the morning by using calciparin (20 U/mL blood) as an anticoagulant. Each blood sample of 20.0 mL, contained in a 50 mL syringe, was immediately treated with the gas mixture composed of a volume of 20.0 mL of a gas mixture composed of O_2_(*∼*96%) and O_3_(*∼*4%), at the ozone concentrations indicated in Table 1. The gas withdrawn in a 20 mL syringe was introduced into the 50 mL syringe containing the blood sample via a multidirectional stopcock. We have previously determined that a rapid rotation of the syringe along its longitudinal axis (about 80 cycles/min) for one minute achieved a complete mixing of the liquid-gas phases with minimal foaming and that, within this period of time, ozone reacts completely with substrates, implying that blood samples receiving ozone react with the ozone dose totally. The pO_2_ reached a value of about 400 mm Hg, while the blood pCO_2_ and pH values did not change. In order to obtain reproducible results, it needs to be emphasized that O_3_ is a very reactive gas meaning that an extremely rapid and precise handling is required. The final gas pressure remained at normal atmospheric pressure. Control sample received only oxygen. Immediately after either the oxygenated or the ozonated samples were centrifuged at 3500 g for 7 minutes, the separated plasma was promptly distributed in cell culture dishes.

### 2.4. Cell culture and incubations

Primary human endothelial cells (HUVECs) were obtained from the neonatal umbilical cord vein as previously described [18]. Endothelial cell growth medium (EGM) with the appropriate supplements and other necessary media were obtained from Clonetics (San Diego, Calif, USA) . For tissue culture procedures, in terms of initiation, subculturing and maintenance, we followed the indications given by Cambrex Inc. (LA, USA). Cells were grown in a humidified incubator at 37°C (95% room air, 5% CO_2,_ pH 7.3) within 3–4 days after growth to confluence. When nitrite measurements were performed, cells were transferred to 24 well (2cm^2^) tissue culture plates and were used at the same cell density and passage number [18].

### 2.5. Biochemical determinations

Hydrogen peroxide (H_2_O_2_) was measured in plasma before and after addition of oxygen-ozone by the enzymatic method described by Green and Hill [19]. Protein thiol groups (PTG) were measured in plasma according to Hu [20] using procedure 1 with 5,5′-dithiobis (2-nitrobenzoic acid, DTNB) dissolved in absolute methanol. The thiobarbituric acid (TBA) assay was carried out in plasma as described by Buege and Aust [21]. Values are expressed as *μ*M of TBA reactive substances (TBARS) relative to a malondialdehyde standard. Production of nitrite concentrations was measured in culture medium supernatants after the addition of predetermined inducers, by using the Griess reagent as previously described [18, 22]. The colored product was spectrophotometrically determined at 538 nm. Nitrite concentration (*μ*M) was determined by comparison with a standard curve made from a solution of NaNO_2_.

### 2.6. Biochemical and immunological assays for endothelial heme oxygenase activity

Confluent HUVECs in 75 cm^2^ flasks were incubated for 18 hours in EGM (alone for the control group) or in the presence of several HO-1 inducers as follows: H_2_O_2_ (from 20 to 200 *μ*M), porcine hematin (2.5 mg/mL saline) HO-1 activity assay. The enzymatic activity was measured by bilirubin generation as described by Motterlini et al. [23] with minimal modifications. HUVECs were grown to confluence in 10 cm tissue culture dishes and then were incubated for 18 hours in EGM (final volume 10 mL, control group) or in the presence of the mentioned inducers. After treatment, cells were washed twice with phosphate-buffered saline, scraped with a rubber policeman, and pelleted at 2500 g for 10 minutes. The cell pellet was suspended in MgCl_2_ (2 mM) phosphate (100 mM) buffer (pH 7.4), frozen at −80°C, thawed three times to break up the cell membrane, and finally sonicated in ice before centrifugation at 18 000 g for 10 minutes at 4°C. 10 *μ*L was taken to determine protein concentration [24]. The final supernatant was added to the reaction mixture (400 *μ*L) containing 3 mg protein of rat liver cytosol prepared from 105 000 g supernatant fraction as a source of biliverdin reductase, 20 *μ*mol/L hemin, 2 mmol/L glucose 6-phosphate, 0.2 units glucose 6-phosphate dehydrogenase, and 0.8 mmol/L *β*-NADPH. The reaction was conducted for 1 hour at 37°C in the dark and terminated by the addition of 1 mL chloroform. The extracted bilirubin was calculated by the difference in absorption between 464 and 530 nm using a quartz cuvette (extinction coefficient, 40 mM^-1^ *⋅* cm^-1^ for bilirubin). HO-1 activity was expressed as picomoles of bilirubin formed per milligram of endothelial cell protein per hour [24].

### 2.7. Western blot analysis

Cells were harvested and lysed in appropriate buffer containing 1% Triton X-100. Equal amounts of proteins, which were determined using a kit from Pierce (Rockford, Ill, USA), were resolved by SDS-polyacrylamide gel electrophoresis, transferred to PVDF filters and subjected to immunoblot using specific antibodies against HO-1 (StressGen Biotechnologies, Canada), Hsp-70 (BD Biosciences, San Jose, CA, USA), and *β*-actin (Cell Signaling Technology, Beverly, MA, USA). Membranes were incubated with an appropriate peroxidase-conjugated secondary antibody, and the antigen-antibody complexes were visualized using an Immuno-Star HRP kit (Bio-Rad Laboratories, Hercules, CA, USA). Nonsaturated-immunoreactive bands were detected with a CCD camera gel documentation system (ChemiDoc XRS, Bio-Rad Laboratories, CA, USA) and then quantitated with Quantity One software (Bio-Rad Laboratories). *β*-actin was used in the same gel to normalize the amounts of total protein present in the samples.

### 2.8. Statistical evaluation

**Figure 1:**
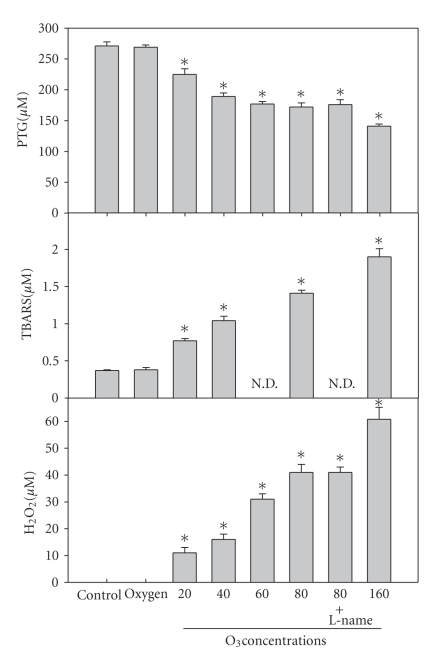
Effect of either oxygenation (O_2_) or ozonation at ozone concentrations of 20, 40, 60, 80, and 160 *μ*g/mL gas per mL of blood (three blood samples of the same donor collected in heparin). To one sample L-NAME, (20 mM) was added. The diagram reports the determinations of protein thiol group (PTG), thiobarbituric acid reactive substances (TBARS), and H_2_O_2_. The statistical significance has been indicated with (*)

Owing to a cell contamination, the average values of only two sets of data are presented in Table 1. Results, although in good agreement, do not allow a statistical analysis. On the other hand, Figure 1 has been statistically evaluated and values are expressed as means and standard deviation. *P* values less than .05 were considered significant and marked with one asterisk.

## 3. RESULTS

Figure 1 shows the modification of the levels of PTG, TBARS, and H_2_O_2_ in relation to the different ozone concentrations. In previous experiments, we have also measured the total antioxidant status of human plasma that transitorily decreases to no less than 60% when the ozone concentration is at 80 *μ*g/mL per mL of blood. PTG show a progressive decrease in relation to the ozone dose and the lowest value (53%) was determined at an ozone concentration of 160 *μ*g/mL per mL of blood. Peroxidation levels were measured as TBARS increased with the ozone dose and their values ensure the ozonation efficiency. Values remained stable in vitro for several hours.

Table 1 shows the results as achieved by evaluating both NO and the HO-1 activity. After 18-hour incubation, NO was measured in the culture supernatants while the supernatants obtained from the cell layers after undergoing the enzyme extraction [23] were incubated for one hour for measuring the generation of bilirubin. H_2_O_2_ induces both NO and HO-1, indirectly measured by the production of bilirubin. As it has been previously observed (23, 25), there is a good relationship between NO and HO-1: addition of L-arginine (20 *μ*M) enhances the production of NO and of HO-1, while the NO-synthase inhibitor L-NAME (20 mM) strongly depresses the release of the messenger and of the enzyme. Without L-arginine, a concentration of 40 *μ*g/mL is more effective than the higher concentration of 80 *μ*g/mL. The same trend has been observed with the use of ozonated human plasma similarly inhibited in the presence of L-NAME.

**Figure 2:**
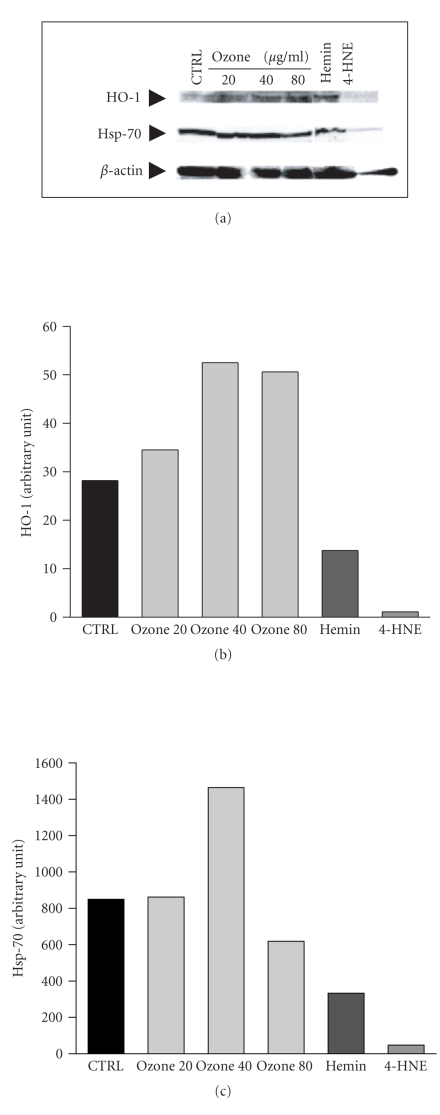
Ozonated human plasma, particularly at the medium ozone concentration of 40 *μ*g/mL per mL of blood activates both HO-1 and Hsp-70 in human endothelial cells. The signals of both protein levels were determined by densitometric analysis of the scanned images. Data are expressed as arbitrary units. One representative Western blot of a typical experiment is shown in the top panel.

Both H_2_O_2_ and ozonated human plasma induce an increased production of NO and bilirubin in a dose-dependent fashion up to noncytotoxic doses. This trend has been confirmed in analyzing, by Western blot, some of the samples; and in Figure 2, we can note that a medium-level plasma ozonation (40 *μ*g/mL) is the most effective in inducing both HO-1 and Hsp-70. Interestingly, either the low or the high doses are less effective. 4-hydroxynonenal was inhibitory probably because it was cytotoxic at the concentration of 10 *μ*M.

## 4. DISCUSSION

Since Maines' extensive review [6] on the heme oxygenase system, its antioxidant and cytoprotective activities have been amply demonstrated in a variety of pathological conditions [26–34]. It is well recognized that HO-1 is induced during oxidative stress due to superoxide , hydroxyl radicals, hypoclorous acid, singlet oxygen, and peroxyl radicals. Furthermore, Motterlini et al. [23] and Wang et al. [25] have clarified that NO and NO-releasing compounds modulate the activity of HO-1. All of these contributions have been important in explaining why some drugs are able to produce anti-inflammatory and antiproliferative effects most likely due to HO-1.

For a long time, we have been involved in clarifying the biological and therapeutic effects of ozone therapy, that in the classical form, consists in exposing for a few minutes a volume (100–225 mL) of human blood to a mixture of oxygen-ozone where the latter gas is only 2–4%. However, its extremely high reactivity causes an acute oxidative stress that activates a number of biochemical pathways [10–15] without any deleterious action on HO-1 blood cells, because the ozone dose is perfectly calibrated against the potent antioxidant capacity of plasma and erythrocytes. While we have shown already the induction of antioxidant enzymes, it remains to demonstrate the production of HO-1. Ozone dissolves in the water of plasma and instantaneously reacts with unsaturated fatty acids and antioxidants and in doing so, disappears but generates H_2_O_2_, a variety of alkenals, and a trace of hemoglobin from no more than 0,7% of the erythrocyte mass. The evaluation of the ozonated plasma has shown the consistency of the ozonation process, particularly in the therapeutic range of 0.42 *μ*mol/mL (20 *μ*g/mL ozone per mL of plasma) up to 1.68 *μ*mol/ mL (80 *μ*g/mL ozone).

In Table 1, we compared the production of NO and HO-1, assessed as bilirubin, because it has been previously ascertained a consequential effect [23, 25]. We have confirmed this relationship except for the highest ozone dose (1.68 *μ*mol/mL) and when the inhibitor of NO synthase was present. Moreover, the optimal induction of HO-1 depends very much from the dose ; in fact, H_2_O_2_ at concentrations of 100 and 200 *μ*g/mL (data not shown) and 4-hydroxynonenal at a concentration of 10 *μ*M have inhibited the induction because they are most likely cytotoxic. It remains unclear why in this experiment heme inhibited the induction of both Hsp-70 and HO-1. Nonetheless, it appears evident, as we have observed also for the induction of antioxidant enzymes, that the optimal induction of HO-1 and Hsp-70, at least in vitro, is achieved by using the medium ozone therapeutic dose (0.84 *μ*mol/mL or 40 *μ*g/mL gas per mL of blood). The biochemical data are consistent with the Western blot analysis and show that both HO-1 and Hsp-70 may play important protective functions in adaptive responses to oxidative stress [35, 36]. In addition, the adaptation to oxidative stress induced by O_3_ treatment has been also shown in animal studies by Zamora et al. and by Ajamieh et al. [37, 38]. This result agrees well with the clinical data because the best therapeutic results have been obtained by starting with low dose up to medium dose.

A final useful comment is that the induction of HO-1 surprisingly has been obtained by administering simvastatin [39], rosuvastatin [40], aspirin [41], and curcumin [42], suggesting the protective activity of this enzyme with other drugs.

## Figures and Tables

**Table 1:** Production of NO (as total nitrite, *μ*M) and HO-1 (as bilirubin, pmol/mg cell protein/1 hour) after stimulation of HUVECs in culture for 18 hours with the indicated inducers.

Inducers	NO	HO-1	As a % increase of HO-1
Medium	1.3	80	100
Medium + O_2_	1.4	86	100
H_2_O_2_ 20 *μ*g/mL	2.3	205	+138
H_2_O_2_ 40 *μ*g/mL	6.7	390	+353
H_2_O_2_ 80 *μ*g/mL	7.5	330	+284
H_2_O_2_ 80 *μ*g/mL + L.Arg	8.1	425	+394
H_2_O_2_ 80 *μ*g/mL + L-NAME	1.2	112	+30
Human Plasma	1.1	127	100
Human Plasma + O_2_	1.2	125	100
Human Plasma + O_2_O_3_ 20 *μ*g/mL	1.7	215	+72
Human Plasma + O_2_O_3_ 40 *μ*g/mL	3.5	290	+132
Human Plasma + O_2_O_3_ 60 *μ*g/mL	4.7	320	+156
Human Plasma + O_2_O_3_ 80 *μ*g/mL	4.2	270	+116
Human Plasma + O_2_O_3_ 80 *μ*g/mL + L-NAME	0.9	149	+19

^1^All values are of the average of two determinations.

## References

[1] Zuckerbraun BS, Billiar TR (2003). Heme oxygenase-1: a cellular hercules. *Hepatology*.

[2] Tenhunen R, Marver H, Pimstone NR, Trager WF, Cooper DY, Schmid R (1972). Enzymatic degradation of heme: oxygenative cleavage requiring cytochrome P-450. *Biochemistry*.

[3] Balla G, Jacob HS, Balla J (1992). Ferritin: a cytoprotective antioxidant stratagem of endothelium. *Journal of Biological Chemistry*.

[4] Barañano DE, Rao M, Ferris CD, Snyder SH (2002). Biliverdin reductase: a major physiologic cytoprotectant. *Proceedings of the National Academy of Sciences of the United States of America*.

[5] Verma A, Hirsch DJ, Glatt CE, Ronnett GV, Snyder SH (1993). Carbon monoxide: a putative neural messenger. *Science*.

[6] Maines MD (1997). The heme oxygenase system: a regulator of second messenger gases. *Annual Review of Pharmacology and Toxicology*.

[7] Abraham NG, Kappas A (2005). Heme oxygenase and the cardiovascular-renal system. *Free Radical Biology and Medicine*.

[8] Bach FH (2005). Heme oxygenase-1: a therapeutic amplification funnel. *The FASEB Journal*.

[9] Wiernsperger NF (2003). Oxidative stress: the special case of diabetes. *BioFactors*.

[10] Bocci V (2002). *Oxygen-Ozone Therapy: A Critical Evaluation*.

[11] Bocci V (2005). *Ozone: A New Medical Drug*.

[12] Bocci V (2006). Scientific and medical aspects of ozone therapy: state of the art. *Archives of Medical Research*.

[13] Bocci V (2006). Is it true that ozone is always toxic? the end of a dogma. *Toxicology and Applied Pharmacology*.

[14] Bocci V (2007). The case for oxygen-ozone therapy. *British Journal of Biomedical Science*.

[15] Bocci V (1996). Does ozone therapy normalize the cellular redox balance? implications for the therapy of human immunodeficiency virus infection and several other diseases. *Medical Hypotheses*.

[16] Calabrese EJ (2005). Paradigm lost, paradigm found: the re-emergence of hormesis as a fundamental dose response model in the toxicological sciences. *Environmental Pollution*.

[17] Keyse SM, Tyrrell RM (1989). Heme oxygenase is the major 32-kDa stress protein induced in human skin fibroblasts by UVA radiation, hydrogen peroxide, and sodium arsenite. *Proceedings of the National Academy of Sciences of the United States of America*.

[18] Valacchi G, Bocci V (2000). Studies on the biological effects of ozone: 11. Release of factors from human endothelial cells. *Mediators of Inflammation*.

[19] Green MJ, Hill HAO (1984). Chemistry of dioxygen. *Methods in Enzymology*.

[20] Hu M-L (1994). Measurement of protein thiol groups and glutathione in plasma. *Methods in Enzymology*.

[21] Buege JA, Aust SD (1978). Microsomal lipid peroxidation. *Methods in Enzymology*.

[22] Moshage H, Kok B, Huizenga JR, Jansen PL (1995). Nitrite and nitrate determinations in plasma: a critical evaluation. *Clinical Chemistry*.

[23] Motterlini R, Foresti R, Intaglietta M, Winslow RM (1996). NO-mediated activation of heme oxygenase: endogenous cytoprotection against oxidative stress to endothelium. *American Journal of Physiology*.

[24] Peterson GL (1977). A simplification of the protein assay method of Lowry *et al.* which is more generally applicable. *Analytical Biochemistry*.

[25] Wang J, Lu S, Moënne-Loccoz P, Ortiz de Montellano PR (2003). Interaction of nitric oxide with human heme oxygenase-1. *Journal of Biological Chemistry*.

[26] Duckers HJ, Boehm M, True AL (2001). Heme oxygenase-1 protects against vascular constriction and proliferation. *Nature Medicine*.

[27] Sato K, Balla J, Otterbein L (2001). Carbon monoxide generated by heme oxygenase-1 suppresses the rejection of mouse-to-rat cardiac transplants. *Journal of Immunology*.

[28] Bach FH (2002). Heme oxygenase-1 as a protective gene. *Wiener Klinische Wochenschrift*.

[29] Durante W (2003). Heme oxygenase-1 in growth control and its clinical application to vascular disease. *Journal of Cellular Physiology*.

[30] Maines MD (2004). The heme oxygenase system: past, present, and future. *Antioxidants and Redox Signaling*.

[31] Takahashi T, Morita K, Akagi R, Sassa S (2004). Heme oxygenase-1: a novel therapeutic target in oxidative tissue injuries. *Current Medicinal Chemistry*.

[32] Camara NOS, Soares MP (2005). Heme oxygenase-1 (HO-1), a protective gene that prevents chronic graft dysfunction. *Free Radical Biology and Medicine*.

[33] Devadas K, Dhawan S (2006). Hemin activation ameliorates HIV-1 infection via heme oxygenase-1 induction. *Journal of Immunology*.

[34] Yamashita K, Öllinger R, McDaid J (2006). Heme oxygenase-1 is essential for and promotes tolerance to transplanted organs. *The FASEB Journal*.

[35] Wong CG, Bonakdar M, Mautz WJ, Kleinman MT (1996). Chronic inhalation exposure to ozone and nitric acid elevates stress-inducible heat shock protein 70 in the rat lung. *Toxicology*.

[36] Valacchi G, van der Vliet A, Schock BC (2002). Ozone exposure activates oxidative stress responses in murine skin. *Toxicology*.

[37] Zamora ZB, Borrego A, López OY (2005). Effects of ozone oxidative preconditioning on TNF-α release and antioxidant-prooxidant intracellular balance in mice during endotoxic shock. *Mediators of Inflammation*.

[38] Ajamieh HH, Menéndez S, Martínez-Sánchez G (2004). Effects of ozone oxidative preconditioning on nitric oxide generation and cellular redox balance in a rat model of hepatic ischaemia-reperfusion. *Liver International*.

[39] Lee T-S, Chang C-C, Zhu Y, Shyy JY-J (2004). Simvastatin induces heme oxygenase-1: a novel mechanism of vessel protection. *Circulation*.

[40] Grosser N, Erdmann K, Hemmerle A (2004). Rosuvastatin upregulates the antioxidant defense protein heme oxygenase-1. *Biochemical and Biophysical Research Communications*.

[41] Grosser N, Abate A, Oberle S (2003). Heme oxygenase-1 induction may explain the antioxidant profile of aspirin. *Biochemical and Biophysical Research Communications*.

[42] Maheshwari RK, Singh AK, Gaddipati J, Srimal RC (2006). Multiple biological activities of curcumin: a short review. *Life Sciences*.

